# Bewertung der ambulanten ärztlichen Schmerztherapie in Deutschland: Ergebnisse einer internetbasierten Querschnittsbefragung unter ambulant tätigen Schmerzmedizinern

**DOI:** 10.1007/s00482-020-00492-8

**Published:** 2020-08-17

**Authors:** V. Kriegisch, B. Kuhn, M.-L. Dierks, J. Achenbach, J. Briest, M. Fink, M. Dusch, V. Amelung, M. Karst

**Affiliations:** 1 Klinik für Anästhesiologie und Intensivmedizin, Schmerzambulanz, Medizinische Hochschule Hannover, Carl-Neuberg-Str. 1, 30625 Hannover, Deutschland; 2 Kassenärztliche Bundesvereinigung, Herbert-Lewin-Platz 2, 10623 Berlin, Deutschland; 3 Institut für Epidemiologie, Sozialmedizin und Gesundheitssystemforschung, Medizinische Hochschule Hannover, Carl-Neuberg-Str. 1, 30625 Hannover, Deutschland; 4 Klinik für Rehabilitationsmedizin, Medizinische Hochschule Hannover, Carl-Neuberg-Str. 1, 30625 Hannover, Deutschland

**Keywords:** Organisation der Schmerztherapie in Deutschland, Unterversorgung chronischer Schmerzpatienten, Mangel an qualifizierten Schmerztherapeuten, Ambulante multimodale Schmerztherapie, Facharzt für Schmerztherapie, Organisation of chronic pain management in Germany, Insufficient medical care of chronic pain patients, Shortage of highly specialized pain therapists, Interdisciplinary chronic pain management on outpatient basis, Board certification for pain management

## Abstract

**Hintergrund:**

Nach den Kriterien der Qualitätssicherungsvereinbarung Schmerztherapie (QSV) nahmen zum Stichtag 31.12.2016 1206 Ärztinnen und Ärzte an der ambulanten Versorgung chronischer Schmerzpatienten teil. Bei in weiten Teilen bestehender Unterversorgung chronischer Schmerzpatienten fehlen Daten zur Einschätzung der ambulanten Schmerztherapie durch die Schmerztherapeuten selbst.

**Methoden:**

In einem Hybrid-Delphi-Verfahren wurde ein Fragebogen zur inhaltlichen, strukturellen und persönlichen Bewertung der ambulanten Schmerztherapie in Deutschland entwickelt.

Mit diesem Instrument wurde eine internetbasierte Querschnittsbefragung von 281 QSV-Schmerzmedizinern aus vier Bundesländern (Berlin, Niedersachsen, Sachsen, Baden-Württemberg) und aller universitären Schmerzambulanzleiter (*n* = 36) in Deutschland durchgeführt.

**Ergebnisse:**

Die Befragung erzielte eine bereinigte Rücklaufquote von insgesamt 35,9 %. Bei den Schmerzambulanzleitern antworteten 66,7 %. Bei 91 % der Befragten lag der Anteil an chronisch Schmerzkranken in der Praxis bei über 70 %. 67,3 % geben an, mit ihrer Praxissituation zufrieden zu sein, auf der anderen Seite äußern 63,4 % ihre Unzufriedenheit mit der aktuellen Organisation der Schmerzmedizin in Deutschland insgesamt. Diese Unzufriedenheit zeigt sich vor allem in Bezug auf die Budgetregelungen (69,3 %), die Kooperation mit Psychotherapeuten (69,3 %) und die interdisziplinäre Vernetzung (50,5 %). Als gute Vorbereitung für den späteren Beruf werden die einjährige Weiterbildung bei einem Weiterbildungsbefugten (87,1 %) und die Teilnahme an dem Kurs „Psychosomatische Grundversorgung“ (90,1 %) bewertet. Vielfältige Freitextkommentare weisen darauf hin, dass die Ausbildung zu kurz und nicht ausreichend sei. Die Mehrheit der Befragten hält es sowohl aus Arztsicht (61,4 %) wie auch aus Patientensicht (54,5 %) für sinnvoll, einen Facharzt für Schmerzmedizin als Versorgungsmodell zu etablieren. 70,8 % der Schmerzambulanzleiter sprechen sich für eigenständige Strukturen mit eigenem Budget aus, 75,0 % geben an, dass ihre Ambulanz unter den aktuellen Bedingungen nicht kostendeckend arbeitet. In Bezug auf die aktuelle Ausbildungssituation berichten nur 39,7 % der QSV-Schmerztherapeuten in der Niederlassung, dass sie auch Ärzte ausbilden, 57,6 % von ihnen planen zudem, ihre Tätigkeit innerhalb der nächsten 10 Jahre aufzugeben.

**Schlussfolgerungen:**

Die mangelnde Eigenständigkeit der Schmerzmedizin und die unzureichend ausgebauten ambulanten Versorgungsnetzwerke tragen dazu bei, dass Schmerztherapeuten mit vielen Aspekten ihrer Tätigkeit unzufrieden sind. Die Etablierung eines Facharztes für Schmerztherapie wird als eine gute Lösung für eine bessere schmerzmedizinische Versorgung und für die Nachwuchsproblematik gesehen.

## Abstract (en)

**Background:**

On 31 December 2016, a total of 1206 physicians participated in the outpatient care of chronic pain patients according to the criteria of a special pain management program (QSV). Because of the largely existing shortage of treatment resources for chronic pain patients, there is a lack of data regarding the evaluation of outpatient pain management by highly specialized pain therapists.

**Methods:**

In a hybrid Delphi procedure, a questionnaire concerning the content, structural and personal assessment of outpatient pain management in Germany was developed. With the help of this instrument, an internet-based cross-sectional survey of 281 QSV pain therapists from four German states (Berlin, Lower Saxony, Saxony, Baden-Württemberg) and of all the heads of university outpatient pain services (*n* = 36) in Germany was conducted.

**Results:**

The adjusted response rate of the survey was 35.9%. The response rate of the heads of university outpatient pain services was 66.7%. In 91% of the respondents the proportion of chronic pain patients in the medical practices was more than 70%. Whereas 67.3% were satisfied with the situation in their medical practice, 63.4% were dissatisfied with the current organizational structure of the pain management in Germany. They expressed their dissatisfaction especially in terms of budgeting regulations (69.3%), the cooperation with psychotherapists (69.3%) and interdisciplinary networking (50.5%). The 1‑year specialist training in pain management (87.1%) and the completion of a psychosomatic basic care course (90.1%) were assessed as a good preparation for the later profession. A multitude of free comments indicated that the pain specialist training is too short and insufficient. Most of the respondents considered the establishment of a specialist board certification for pain management more suitable from the perspective of physicians (61.4%) and the patients (54.5%). Of the heads of university outpatient pain services 70.8% expressed the wish for autonomous structures with separate budgets and 75.0% indicated that under the current conditions their outpatient pain services are not working profitably. Only 39.7% of the QSV pain therapists provided fellowship training for physicians and 57.6% were planning to retire during the next 10 years.

**Conclusion:**

Highly specialized pain therapists are dissatisfied because of the lack of independence of the organizational structure of pain management care and the insufficient interdisciplinary network in outpatient pain management. A possible solution for a better pain management care and the recruitment problems may be the establishment of a board certification for pain management.

## Einleitung

Chronische Schmerzen treten häufig auf. In einer Querschnittsbefragung in Deutschland berichteten 27 % der Befragten über chronische Schmerzen in Bezug auf die Dauer ihrer Beschwerden [1]. Allein für die Versorgung von Patienten mit chronischen Rückenschmerzen fallen in Deutschland jährlich direkte und indirekte Kosten von ca. 50 Mrd. € an, wovon etwa zwei Drittel auf die Kosten für Frühberentung entfallen [2] und ein nicht unerheblicher Teil für die Schmerztherapie.

70 % der chronischen Schmerzpatienten werden durch Allgemeinärzte, 27 % durch Orthopäden und nur 2 % durch Schmerztherapeuten behandelt. Lediglich 10 % der deutschen Schmerzpatienten haben nach dem 2011 erschienen Health-Technology-Assessment(HTA)-Bericht bislang jemals einen Schmerztherapeuten aufgesucht [3], dies zeigt deutlich die Unterversorgung, die auch in 2020 noch virulent sein dürfte.

Der 99. Deutsche Ärztetag hatte bereits 1996 die Einführung einer fachgebietsbezogenen Zusatzbezeichnung „spezielle Schmerztherapie“ beschlossen, 2005 wurde die Qualitätssicherungsvereinbarung (QSV) nach § 135 Abs. 2 SGB V eingeführt. Für die Zusatzbezeichnung „spezielle Schmerztherapie“ ist unter anderem eine 12-monatige Weiterbildung bei einem entsprechenden Weiterbildungsermächtigten erforderlich. Zur Teilnahme an der QSV ist zusätzlich die erfolgreich abgeschlossene Weiterbildung „Psychosomatische Grundversorgung“ notwendig sowie eine Prüfung vor der Schmerztherapie-Kommission der Kassenärztlichen Vereinigung.

Durch die Teilnahme an der QSV ist es den Schmerztherapeuten möglich, entsprechende höherwertige Abrechnungsziffern zu nutzen, die dem erhöhten Zeitaufwand für Anamnese, Begleitung und umfassende ärztliche Versorgung der Schmerzpatienten mit oftmals komplexen Krankheitsbildern gerecht werden. Somit wird neben der Sicherung der Qualität ein finanzieller Anreiz geschaffen, um die Versorgung der chronisch schmerzkranken Patienten zu gewährleisten und die Attraktivität der Schmerzmedizin zu erhöhen.

Zum Stichtag 31.12.2016 nahmen in Deutschland 1206 Ärztinnen und Ärzte an der QSV teil [4]. Im Bundesdurchschnitt versorgt ein Schmerztherapeut 70.000 Einwohner, Niedersachsen ist das Schlusslicht mit einem Schmerztherapeuten für 152.500 Einwohner [5]. Etwa die Hälfte aller ambulant tätigen Schmerztherapeuten beendet voraussichtlich die Tätigkeit innerhalb der nächsten 5 Jahre [4]. Bei Hausärzten beträgt diese Quote nur etwa 20 % [6].

Die Vergütungssituation ist uneinheitlich. Die Budgets für Arznei- und Heilmittel haben sich lange an den jeweiligen Fachgebieten orientiert und erst jüngst am Vergleich innerhalb der QSV-Gruppe.

Die Komplexität der Schmerzerkrankung verlangt in der Regel eine multimodale Therapie, die in vielen ambulanten Netzwerken auch angeboten wird, aber aus Sicht der Kostenträger nur für den stationären Sektor vorgesehen ist.

Die im Folgenden präsentierte Querschnittsbefragung wurde initiiert, um von den Personen, die täglich chronische Schmerzpatienten versorgen, zu erfahren, wie zufrieden sie mit ihrer beruflichen Situation sind und welche Änderungen in der Organisation der Schmerzmedizin sie sich für ihr eigenes berufliches Handeln und für eine bessere Versorgungssituation chronischer Schmerzpatienten wünschen. Ein zusätzliches Augenmerk wurde auf die universitären Schmerzambulanzen gerichtet. Denn dort entscheidet sich vielfach, ob sich ausreichend viele ärztliche Interessenten finden und ausbilden lassen, um auch in Zukunft eine qualitativ hochwertige Schmerztherapie in Deutschland zu gestalten.

## Methoden

Von Dezember 2017 bis Januar 2018 erfolgte eine internetbasierte, standardisierte Befragung von Schmerzmedizinern aus vier verschiedenen Bundesländern (siehe unten). Dabei wurden ausschließlich diejenigen Ärztinnen und Ärzte einbezogen, die an der QSV Schmerztherapie teilnehmen, und alle Schmerzambulanzleiter der universitären Schmerzambulanzen.

Über die Arztauskunft und die Suchoption „Zusatzbezeichnungen: spezielle Schmerztherapie“ und „genehmigungspflichtige Leistungen: Schmerztherapie“ konnten in einem ersten Schritt in 10 Bereichen der Kassenärztlichen Vereinigung (KV) 665 QSV-Ärztinnen und -Ärzte identifiziert werden. Um eine für Gesamtdeutschland möglichst aussagekräftige Auswahl zu erreichen, wurden aus dieser Liste die nachfolgend genannten KV-Bereiche nach den folgenden Kriterien ausgewählt:

- Berlin als bevölkerungsreichste Stadt und Stadtstaat
- Sachsen als eines der fünf „neuen Länder“ in der ländlich geprägten Region im Osten
- Niedersachsen als zweitgrößtes Flächenland im mittleren Nordwesten
- Baden-Württemberg als drittgrößtes Bundesland im Südwesten

In diesen Bundesländern entsprachen dann 281 Ärztinnen und Ärzte den Auswahlkriterien.

Der Fragebogen wurde in einem mehrstufigen Vorgehen im Sinne eines Hybrid-Delphi-Prozesses in einer Kombination aus Fokus- und Nominalgruppentechnik und Delphi-Methode inhaltlich und formal entwickelt [7]. In der Fokusgruppentechnik (FGT) werden Fragestellung und der theoretische und konzeptionelle Rahmen eines Forschungsvorhabens diskutiert. Hierzu wurde die Expertise des Doktorandenkolloquiums des Instituts für Epidemiologie, Sozialmedizin und Gesundheitssystemforschung der MHH genutzt, das sich aus Mitarbeitern des Instituts und Doktoranden zusammensetzt. Dort wurde das Vorhaben in mündlicher und schriftlicher Form vorgestellt. Diskussionsbeiträge wurden in den Fragebogen aufgenommen und in weiteren Treffen diskutiert. In der Nominalgruppentechnik (NGT) erläutern Experten konkrete und typische Vorgehensweisen auf dem Feld ihrer Expertise. Hierzu wurde von den Mitarbeitern des Instituts für Epidemiologie, Sozialmedizin und Gesundheitssystemforschung der MHH beispielhaft Fragebögen zu anderen Themen vorgestellt. Vor- und Nachteile in Bezug auf die Entwicklung des eigenen Fragebogens wurden innerhalb der Experten und mit dem gesamten Forschungsteam diskutiert. In der Delphi-Methode werden die (vorläufigen) Ergebnisse der methodischen Vorgehensweise des Forschungsvorhabens den bis dahin nicht an der Diskussion beteiligten unabhängigen Experten vorgestellt. Aus mindestens zwei Runden aus Vorstellen und Beurteilung durch die befragten Experten können sich weitere Veränderungen der methodischen Vorgehensweise ergeben. Unser (vorläufiger) Fragebogen wurde unabhängigen Experten vorgestellt. Einzelne Kritikpunkte wurden aufgenommen und haben zur weiteren Verbesserung beigetragen. Der Prozess der Entwicklung des Fragebogens bis zum Beginn der Befragung nahm einen Zeitraum von ca. 1,5 Jahren in Anspruch.

Das Erhebungsinstrument enthielt abschließend 40 Fragen, die die Zufriedenheit der Befragten mit unterschiedlichen Aspekten der Schmerzversorgung mittels vier- bis fünfstufiger Antwortskalen erfassten, in Ergänzung waren (halb-)offene Fragen integriert. Der Fragebogen wurde über die internetbasierte Fragebogenplattform SurveyMonkey zur Verfügung gestellt und konnte hier von den Teilnehmenden anonym beantwortet werden.

Die Fragebögen und das Begleitschreiben wurden von der Ethikkommission der Medizinischen Hochschule Hannover als ethisch und (datenschutz-)rechtlich unbedenklich eingeschätzt (Prüfungsnummer: 3489-2017).

Die Auswertung der Daten erfolgte unter Verwendung der Software SPSS 26.0 (IBM Corp., IBM SPSS Statistics for MAC, Version 26.0. Armonk, NY; USA). Freitextantworten wurden inhaltsanalytisch aufbereitet und offen codiert [8].

Die Analyse erfolgte zunächst deskriptiv. Gruppenunterschiede wurden, abhängig vom Skalenniveau, mithilfe von zweiseitigen χ^2^-Tests und t‑Tests auf statistische Signifikanz geprüft. Zur Testung von Zusammenhängen zwischen zwei kontinuierlichen Merkmalen wurden nichtparametrische Korrelationstests durchgeführt und der Korrelationskoeffizient Kendalls Tau‑b (τ_b_) angegeben. Einflussfaktoren auf die Zufriedenheit der Befragten mit der von ihnen angebotenen Schmerztherapie wurden mittels logistischer Regressionsanalyse geprüft. Die im Modell als Prädiktoren eingeschlossenen Variablen wurden zuvor in univariaten Analysen identifiziert.

## Ergebnisse

**Figure 1:**
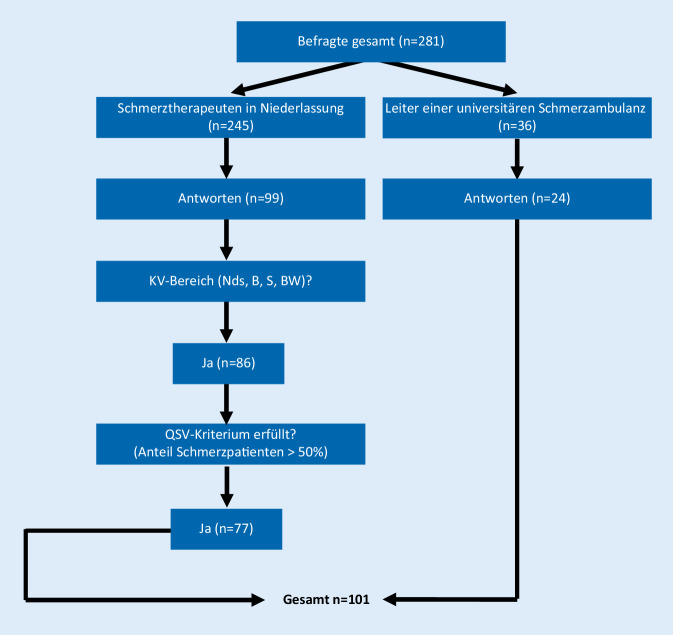

Von insgesamt 281 befragten Personen waren 245 niedergelassene Schmerztherapeuten und 36 Leiter einer Schmerzambulanz. Im niedergelassenen Bereich haben 99 Personen geantwortet, bei den Leitern der universitären Schmerzambulanzen 24 Personen. Unter den niedergelassenen Schmerztherapeuten erfüllten 77 alle Befragungskriterien und gelangten in die Endauswertung (Abb. 1). Insgesamt konnte eine bereinigte Rücklaufquote von 35,9 % erreicht werden (*n* = 101 von 281; Abb. 1).

Sofern keine signifikanten Unterschiede im Antwortverhalten zwischen den niedergelassenen Schmerztherapeuten und den universitären Schmerzmedizinern detektiert werden konnten, wurden die Ergebnisse des Gesamtkollektivs dargestellt. Bei signifikanten Unterschieden zwischen den beiden Gruppen wurde das Ergebnis für jede Gruppe einzeln dargestellt.

Bei den niedergelassenen QSV-Schmerztherapeuten betrug die Rücklaufquote insgesamt 35,1 % (*n* = 86 von 245), die zwischen den KV-Bereichen stark differierte:

- Niedersachsen: 71,8 % (*n* = 32 von 45)
- Berlin: 35,7 % (*n* = 10 von 28)
- Baden-Württemberg: 33,6 % (*n* = 40 von 119)
- Sachsen: 7,6 % (*n* = 4 von 53)

Bei den Leitern der universitären Schmerzambulanzen zeigte sich mit 66,7 % (*n* = 24 von 36) ein ähnlich hohes Antwortverhalten wie bei den Schmerztherapeuten im Bereich der KV Niedersachsen. Der Fragebogen wurde von Schmerzambulanzleitern aus 13 verschiedenen Bundesländern beantwortet.

### Merkmale der Befragten

Das Durchschnittsalter aller Befragten beträgt 55,1 Jahre (Standardabweichung [SD] = 6,9), zwei Drittel sind männlich. Der überwiegende Anteil der Teilnehmer hat als Facharzthintergrund Anästhesiologie (88,1 %), knapp 9 % einen Facharzt der Allgemeinmedizin. Der Anteil an chronisch schmerzkranken Patienten in der jeweiligen Praxis oder Ambulanz liegt bei 9 % der Befragungsteilnehmer zwischen 50 und 69 %, bei 25 % der Befragten zwischen 70 und 89 % und bei 66 % bei ≥90 %. Somit erfüllt die Mehrheit (91 %) aller Befragten die Kriterien einer schmerztherapeutischen Einrichtung. Knapp die Hälfte aller Befragten gibt an, pro Quartal durchschnittlich 300 bis 450 chronische Schmerzpatienten zu behandeln, gut ein Drittel behandelt weniger als 300 dieser Erkrankten. Bei etwa der Hälfte der Praxen liegt die Quote der Neuvorstellungen pro Quartal bei 20 %, bei 10 % der Praxen oder Ambulanzen bei über 50 %.

### Zufriedenheit mit der Versorgungssituation

67,3 % aller Befragten sind mit der Schmerztherapie in ihrer Praxis oder Ambulanz zufrieden bzw. eher zufrieden. Im Rahmen einer logistischen Regressionsanalyse wurde untersucht, welche Faktoren die Chance auf diese Zufriedenheit potenziell beeinflussen (Tab. 1). Es zeigt sich, dass die Gesamtzufriedenheit in hohem Maße mit der Zufriedenheit mit den therapeutischen Möglichkeiten (Odds Ratio [OR] = 3,76) und mit den personellen Strukturen in der Praxis/Ambulanz korreliert (OR = 3,46). Für die Variablen Zufriedenheit mit der Zeit für Patienten, dem Einkommen sowie der Zusammenarbeit mit Physiotherapeuten und Psychologen kann hingegen kein signifikanter Zusammenhang dargestellt werden. Befragte, die angeben, besonders auf das Verordnungsvolumen zu achten, haben hingegen eine signifikant geringere Chance, mit der Schmerztherapie in der eigenen Praxis zufrieden zu sein (OR = 0,36).

**Table 1:** 

	Zufriedenheit Schmerztherapie in eigener Praxis *Nagelkerkes R*^*2*^ *=* *0,53*	
Variablen	Odds Ratio (95 %-KI)	Sig.
Geschlecht: männlich	0,34 (0,06; 1,85)	0,210
Alter	0,91 (0,82; 1,01)	0,070
Tätigkeit an Universität	1,15 (0,15; 8,78)	0,895
Zufriedenheit mit therapeutischen Möglichkeiten	*3,76 (1,40; 10,07)*	0,008
Zufriedenheit mit Zeit für Patienten	1,34 (0,54; 3,31)	0,524
Zufriedenheit mit personellen Strukturen in Praxis/Ambulanz	*3,46 (1,48; 8,06)*	0,004
Enge Zusammenarbeit mit Psychologen	0,41 (0,15; 1,13)	0,083
Enge Zusammenarbeit mit Physiotherapeuten	2,38 (0,76; 7,41)	0,136
Ich achte darauf, das Verordnungsvolumen meiner Fachgruppe nicht zu überschreiten	*0,36 (0,14; 0,92)*	0,033
Ich achte darauf, das Regressrisiko zu minimieren	0,96 (0,39; 2,34)	0,931
Zufriedenheit mit dem Einkommen	1,94 (0,97; 3,89)	0,062
Würden Sie unter den aktuellen Bedingungen erneut Ihre aktuelle Berufssituation wählen?	1,20 (0,61; 2,39)	0,601

*n* = 84; vom Maximum abweichendes *n* je nach Vollständigkeit des Antwortverhaltens; *kursiv* = signifikanter Einfluss (*p* < 0,05)

Deutlich unzufriedener (63,4 %) zeigen sich die Befragten der Gesamtstichprobe mit der Organisation der Schmerztherapie innerhalb Deutschlands. Bei den Schmerzambulanzleitern wird diese Unzufriedenheit sogar von 70,8 % der Umfrageteilnehmer geäußert. Nur 53,5 % aller Umfrageteilnehmer würden unter den aktuellen Bedingungen die aktuelle Berufssituation erneut wählen (52,1 % der niedergelassenen Schmerztherapeuten und 66,7 % der universitären Schmerztherapeuten).

Gut zwei Drittel aller Befragten (69,3 %) sind zudem mit der Budgetregelung (eher) unzufrieden. In den durchgeführten Korrelationsanalysen zeigen sich kleine, jedoch statistisch signifikante Zusammenhänge zwischen dieser Unzufriedenheit und der Überweisung der Patienten an Kooperationspartner für notwendige Verordnungen von Medikamenten und Hilfsmitteln (τ_b_ = 0,23; *p* = 0,007) sowie einem weniger rationalen und leitliniengerechten Vorgehen bei der Verordnung von Hilfsmitteln in Abhängigkeit des Schmerzbilds (τ_b_ = -0,18; *p* = 0,032).

Nur 36,6 % der QSV-Schmerztherapeuten der Gesamtstichprobe sind mit ihrem Einkommen zufrieden, bei den niedergelassenen QSV-Schmerztherapeuten berichten dies 34,2 %, bei den Umfrageteilnehmern der Schmerzambulanzen 45,8 %.

### Zusammenarbeit mit anderen Fachgruppen

50,5 % aller Befragten geben an, dass sie ihren Patienten in Bezug auf eine enge und zeitnahe Zusammenarbeit mit anderen Fachgruppen nicht gerecht werden können, auch wenn 66,3 % grundsätzlich eng mit Psychologen und 76,2 % mit Physiotherapeuten zusammenarbeiten. Die Qualität dieser Zusammenarbeit wird jedoch unterschiedlich bewertet (Tab. 2 und 3).

**Table 2:** 

		Antwortmöglichkeiten (%)		
	*n*	(Eher) unzufrieden	Weder noch	(Eher) zufrieden
Termine	101	69,3	11,9	18,8
Diagnostik	101	46,5	14,9	38,6
Therapieergebnis	101	28,7	29,7	41,6
Austausch	100	40,6	20,8	37,6

**Table 3:** 

		Antwortmöglichkeiten (%)		
	*n*	(Eher) unzufrieden	Weder noch	(Eher) zufrieden
Termine	100	14,9	10,9	73,3
Diagnostik	100	23,8	23,8	51,5
Therapieergebnis	100	18,8	16,8	63,4
Austausch	99	28,7	18,8	50,5

Eine weitere Frage in der Erhebung bezog sich auf die Kooperation mit Schmerzkliniken. Diese wird von 73,3 % aller Befragten als gut beschrieben. Nur 9,9 % sind mit dieser Zusammenarbeit unzufrieden, 10,9 % sind diesbezüglich unentschieden, 3,0 % geben an, keine Kooperation zu haben.

### Ausbildung/Nachwuchs

Die einjährige Weiterbildung bei einem Weiterbildungsermächtigten zum Erwerb der Zusatzbezeichnung „spezielle Schmerzmedizin“ bewerten 87,1 % aller Befragten als Ausbildungskonzept für sich selbst und für andere als positiv. Von insgesamt 19 Anmerkungen im Freitext weisen 10 direkt oder indirekt darauf hin, dass die Ausbildung zu kurz und nicht ausreichend ist. Insbesondere psychosomatische/psychiatrische Inhalte werden vermisst.

Die Absolvierung des Kurses „Psychosomatische Grundversorgung“ sehen 90,1 % aller Befragten in Bezug auf die Relevanz für die berufliche Tätigkeit positiv. 16 von 19 Anmerkungen im Freitext stufen den Kurs „Psychosomatische Grundversorgung“ als unabdingbar ein, 10 empfinden die Kursinhalte in Bezug auf die schmerztherapeutische Arbeit als nicht ausreichend.

Die Teilnahme an 8 Schmerzkonferenzen pro Jahr befürworten 84,2 % aller Befragten.

Bei der Frage, wie viele Ärzte aktuell in „spezieller Schmerztherapie“ von den Befragten ausgebildet werden, differieren die Angaben der Schmerzambulanzleiter und der ambulant tätigen Kollegen. 4,2 % der Ambulanzleiter bilden derzeit keine Ärzte aus oder haben keine Weiterbildungsermächtigung. Im niedergelassenen Bereich trifft dies auf 50,7 % der Befragten zu (*p* < 0,001). Mit 56,3 % wird diese Angabe am häufigsten von Ärzten aus Niedersachsen gemacht.

87,5 % der Schmerzambulanzleiter und 39,7 % der niedergelassenen Kollegen geben an, 1–3 Ärzte auszubilden.

29,2 % der Schmerzambulanzleiter und 57,6 % der QSV-Therapeuten im niedergelassenen Sektor planen, in den nächsten 10 Jahren die Berufstätigkeit aufzugeben (*p* = 0,003).

Die Wahrscheinlichkeit, dass sich nach der Berentung ein Nachfolger für den Standort finden wird, schätzen die Schmerzambulanzleiter mit durchschnittlich 87,8 % ein, die QSV-Therapeuten in der Niederlassung mit durchschnittlich 51,0 % (*p* < 0,001).

### Veränderungswünsche

Den Befragten wurden drei mögliche Versorgungsmodelle genannt, die sie hinsichtlich ihrer Eignung für sich selbst und für ihre Patienten einschätzen sollten:

- Erhaltung des Status quo
- Stärkung der schmerzmedizinischen Kompetenz der Allgemeinmedizin
- Einführung eines eigenen Facharztes für Schmerzmedizin

Die Ergebnisse für die niedergelassenen Schmerztherapeuten zeigen Tab. 4 und 5.

**Table 4:** 

		Antwortmöglichkeiten (%)		
	*n*	(Eher) nicht geeignet	Teils, teils	(Eher) geeignet
Erhaltung Status quo	73	30,1	30,1	39,7
Stärkung Allgemeinmedizin	73	31,5	27,4	41,1
Einführung eigener Facharzt	73	27,4	8,2	64,4

**Table 5:** 

		Antwortmöglichkeiten (%)		
	*n*	(Eher) nicht geeignet	Teils, teils	(Eher) geeignet
Erhaltung Status quo	73	35,6	31,5	32,9
Stärkung Allgemeinmedizin	73	26,0	24,7	49,3
Einführung eigener Facharzt	73	23,3	19,2	57,5

Etwas geringer ausgeprägt als die niedergelassenen Schmerztherapeuten favorisieren die 24 universitären Schmerztherapeuten, die diese Frage beantwortet haben, für sich selbst zu 50,0 % (vs. 64,4 % bei nichtuniversitären Schmerztherapeuten, *p* = 0,145) und in Bezug auf ihre Patienten zu 45,8 % (vs. 57,5 % bei nichtuniversitären Schmerztherapeuten, *p* = 0,005) die Einführung eines eigenen Facharztes für Schmerzmedizin. Die vollständige Auswertung dieser Frage findet sich in Tab. 6 und 7.

**Table 6:** 

		Antwortmöglichkeiten (%)		
	*n*	(Eher) nicht geeignet	Teils, teils	(Eher) geeignet
Erhaltung Status quo	24	16,7	37,5	45,8
Stärkung Allgemeinmedizin	24	20,8	41,7	37,5
Einführung eigener Facharzt	24	45,8	4,2	50,0

**Table 7:** 

		Antwortmöglichkeiten (%)		
	*n*	(Eher) nicht geeignet	Teils, teils	(Eher) geeignet
Erhaltung Status quo	23	25,0	29,2	45,8
Stärkung Allgemeinmedizin	23	16,7	45,8	37,5
Einführung eigener Facharzt	23	50,0	4,2	45,8

Mit einer offenen Frage wurde gefragt, was die Schmerzmediziner an ihrer beruflichen Situation gerne ändern würden. Bei insgesamt 62 Anmerkungen in der Gesamtstichprobe wird besonders häufig genannt:

- Weniger Bürokratie und bessere Finanzierung und dadurch mehr Zeit für Patienten (*n* = 36)
- Stärkere Vernetzung/Interdisziplinarität (*n* = 14)
- Bessere Realisierbarkeit der Ausbildung von angehenden Schmerzmedizinern (*n* = 9)
- Mehr Wertschätzung innerhalb und außerhalb des Facharzthintergrunds (*n* = 8)

### Schmerzambulanzleiter

In die Befragung waren 3 Fragen ausschließlich an die Leiter der Schmerzambulanzen integriert, von denen 95,8 % (*n* = 23) den Facharzthintergrund Anästhesiologie und 4,2 % (*n* = 1) Neurologie aufwiesen.

Mit der Lehre verbringen Schmerzambulanzleiter durchschnittlich 4,4 (SD = 6,4) Stunden pro Woche, auf administrative Aufgaben entfallen durchschnittlich 8,5 (SD = 8,4) Wochenstunden und mit Forschung oder Gutachtertätigkeit werden im Durchschnitt 6,1 (SD = 8,3) Stunden pro Woche verbracht.

Zur Kostensituation der jeweiligen Schmerzambulanz weisen 75,0 % (*n* = 18) darauf hin, dass ihre Ambulanz nicht kostendeckend sei, nur 12,5 % (*n* = 3) sehen in ihrer Ambulanz eine ausgeglichene finanzielle Situation.

70,8 % (*n* = 17) sprechen sich für eigenständige Strukturen mit eigenem Budget aus, während 12,5 % (*n* = 3) dem ablehnend gegenüberstehen.

## Diskussion

### Diskussion der Ergebnisse

Die Mehrheit aller befragten Schmerztherapeuten zeigt sich mit dem eigenen beruflichen Umfeld zufrieden, nicht aber mit der Organisation der Schmerzmedizin in Deutschland. Unzufriedenheit zeigt sich insbesondere bei der Beurteilung der Qualität und Quantität der Vernetzung mit psychologischen Therapieansätzen. Es werden Ausbildungsmängel genannt verbunden mit zu geringem Nachwuchs. Im niedergelassenen Bereich sehen es nur 51 % als wahrscheinlich an, dass sie einen Schmerztherapeuten für ihre Praxis finden, wenn sie ihre Berufstätigkeit aufgeben. Nur ca. 40 % der niedergelassenen Schmerzmediziner bilden aus, im Gegensatz zu ca. 90 % im universitären Bereich. Drei Viertel der universitären Ambulanzen können aber unter den aktuellen Bedingungen nicht wirtschaftlich arbeiten, weshalb sich ca. 71 % der Universitätsangehörigen für eigenständige Strukturen mit eigenen Finanzmitteln aussprechen. Fünfzig Prozent der universitären Schmerzmediziner und 64,4 % der niedergelassenen Schmerztherapeuten befürworten die Schaffung eines eigenen Facharztstatus.

Im Gegensatz zur Einschätzung der QSV-Schmerztherapeuten zeigten in einer 2016 durchgeführten Umfrage bei ca. 10.000 niedergelassenen und angestellten ambulant tätigen Ärzten in Deutschland mehr als 90 % eine gute Zufriedenheit mit ihrer Arbeit (QSV: 67,3 %), 85 % würden den Beruf wieder ergreifen (QSV: 54 %; [9]). 70 % der Hausärzte und 64 % der Fachärzte waren mit ihrem Monatseinkommen zufrieden (QSV: 36,6 %; [9]). 24 % planten, in den nächsten Jahren ihre Praxis abzugeben (QSV: 57,6 %), 44 % hatten einen Weiterbildungsassistenten beschäftigt (QSV: 39,7 %; [9]). Die Prognosen in Bezug auf den schmerztherapeutischen Nachwuchs sind besonders bedenklich, weil die Zahl der chronischen Schmerzpatienten aufgrund der demografischen Entwicklung in den nächsten Jahren voraussichtlich weiter zunehmen wird [10].

In einer Umfrage des BVSD, die 2019 publiziert worden ist [4], wurden 212 Ärzte aus ganz Deutschland befragt. Es waren Mitglieder aller Kassenärztlichen Vereinigungen (KV) vertreten. Am zahlreichsten beteiligten sich Mitglieder der KV Bayern (*n* = 29), KV Baden-Württemberg (*n* = 24), KV Sachsen (*n* = 24) und KV Niedersachsen (*n* = 19). 80 % der Umfrageteilnehmer waren Anästhesisten, 88 % nahmen an der QSV teil. In eigener Praxis oder in einer Gemeinschaftspraxis arbeiteten 47 %, in einem medizinischen Versorgungszentrum (MVZ) 12 %, als Krankenhausärzte persönlich ermächtigt waren 13 % und nur 2 % waren an einer Universitätsklinik beschäftigt. Nach welcher Strategie die Adressen der Befragten ermittelt worden sind, wurde nicht offengelegt. In dieser Umfrage stand nicht die Zufriedenheit der Befragten im Vordergrund, sondern die Frage nach der Auswirkung der regulatorischen und wirtschaftlichen Rahmenbedingungen auf die Versorgungssituation von chronischen Schmerzpatienten. Im Fazit kamen die Autoren zu dem Schluss, dass zu wenig Schmerzmediziner existieren, um den Versorgungsbedarf zu decken, dass sich diese Situation durch den nahenden Ruhestand von mehr als der Hälfte der heute ambulant tätigen Schmerzmediziner noch zuspitzen wird und dass für eine positive Einwirkung auf diese Situation strukturelle Veränderungen notwendig sind [4]. Zu ähnlichen Schlussfolgerungen kam bereits der in 2011 publizierte HTA-Bericht, der die Versorgungssituation in der Schmerztherapie in Deutschland im internationalen Vergleich hinsichtlich Über‑, Unter- oder Fehlversorgung untersucht hat [3]. Es wurde festgestellt, dass eine Unterversorgung an schmerztherapeutischen Einrichtungen besteht und insbesondere auch eine Unterversorgung im Einsatz psychotherapeutischer Verfahren und von Psychologen bei der Schmerzversorgung zu beobachten ist [3]. Neben Verbesserungen der Aus‑, Fort- und Weiterbildung für alle beteiligten Berufsgruppen wurde empfohlen, das Berufsfeld der Schmerztherapeuten einheitlich zu regeln [3]. Um eine fachübergreifende Versorgung chronischer Schmerzpatienten zu gewährleisten, wurde die Einführung des Facharztes für Schmerzmedizin für sinnvoll erachtet [3]. Darüber hinaus wurde empfohlen, die Schmerzmedizin in die Approbationsordnung aufzunehmen, was 2012 als prüfungsrelevantes Querschnittsfach 14 (Q14) auch geschah. Auf sehr hohem Niveau wurden Lehrmaterialien, Unterrichtsgestaltungen und Prüfungsformate entwickelt. Nun sehen die Planungen der aktuellen Approbationsordnung 2020 vor, Q14 als nicht mehr lehr- und prüfungsrelevant einzustufen [11], wogegen in dem noch anhaltenden Diskussionsprozess von der Deutschen Schmerzgesellschaft e. V. Einspruch erhoben worden ist^1^. Ein stärkeres Profil der Schmerzmedizin im Fächerkanon, z. B. durch Einführung eines eigenständigen Facharztstatus, könnte die korrekte Behandlung der schmerzmedizinischen Inhalte in der Ausbildung von Studierenden verbessern und stabilisieren.

Aus den Antworten lassen sich Schlussfolgerungen ableiten, die dazu beitragen könnten, die schmerzmedizinische Versorgungssituation zu verbessern. Aus Sicht der hochgradig spezialisierten QSV-Schmerztherapeuten wird insbesondere eine größere Eigenständigkeit und stärkere Abgrenzung gegenüber der jeweiligen Mutterdisziplin gefordert. Dies gilt auch für die Intensivierung der schmerztherapeutischen Ausbildung. Über die Implementation eines eigenen Facharztes in Schmerztherapie wird in Deutschland seit 2001 in einigen Fachverbänden diskutiert [12]. Argumente hierfür sind im Folgenden aufgeführt:

- Schärfung des Profils
- Mehr Wertschätzung im Medizinsystem
- Sicherung der Finanzierung bei hohem Zeitbedarf und aufwendiger Vernetzung
- Sicherung der Bedarfsplanung
- Mehr Attraktivität für Ausbildung und Nachwuchs

Tatsächlich haben die bisherigen Maßnahmen (z. B. Zusatzbezeichnung „spezielle Schmerztherapie“, Einführung von QSV) nicht dazu beigetragen, dass flächendeckend eine qualitativ und quantitativ gute und zukunftsfähige schmerzmedizinische Versorgung in Deutschland entstanden ist [4, 12, 13]. Neuere Initiativen zur Stabilisierung und Verbesserung des Versorgungsauftrags sind:

- Abgestufte Versorgungsbereiche (Stärkung der schmerzmedizinischen Kompetenz in der Allgemeinmedizin) entsprechend eines Entwurfs der Kassenärztlichen Bundesvereinigung [14]
- „Förderung und Sicherstellung einer flächendeckenden spezifischen schmerzmedizinischen Behandlung von Patienten mit chronischen Schmerzen in der ambulanten vertragsärztlichen Versorgung durch ein interdisziplinäres Team“ entsprechend einem Vorschlag der Kassenärztlichen Bundesvereinigung [14]
- Orientierung der Arznei- und Heilmittelbudgets innerhalb der QSV-Vergleichsgruppe (nicht orientiert an der jeweiligen Facharztgruppe, z. B. Anästhesiologie)
- Empfehlung einer separaten Planung von Anästhesisten und Schmerztherapeuten zur Weiterentwicklung der Bedarfsplanung i. S. d. §§ 99 ff. SGB V durch den Gemeinsamen Bundesausschuss [15]

Ähnlich wie die bisherigen Maßnahmen ist keiner dieser Ansätze grundsätzlich mit einer strukturellen Veränderung des Fachbereichs Schmerzmedizin verbunden. Aus diesem Grund muss gefragt werden, ob es durch diesen Maßnahmenkatalog zu einer nachhaltigen Stabilisierung in der Versorgung von chronischen Schmerzpatienten kommt.

Unter der grundsätzlichen Annahme, dass es nicht genügend Interessenten für einen Facharzt für Schmerzmedizin geben würde, da dessen inhaltliche Attraktivität nicht mit etablierten Fachgebieten konkurrieren könnte [16], wurde als alternative Lösung die Stärkung der schmerzmedizinischen Kompetenz in der Primärversorgung in Kombination mit der Schaffung von Struktur- und Qualitätskriterien für schmerzmedizinische Einrichtungen genannt [17]. Ob solche Kriterien tatsächlich die Qualität und vor allem die Quantität verbessern würden, ist anzuzweifeln. Wie aus der hier vorgelegten Befragung deutlich geworden ist, sind der Schlüssel für eine gute Schmerztherapie die fachliche und persönliche Qualität des jeweiligen Schmerztherapeuten und die Möglichkeit einer engen Zusammenarbeit mit anderen Behandlungsdisziplinen. Multimodale Ansätze können erst vor einem solchen Hintergrund tragfähig werden. Es sind aber genau diese multimodalen Ansätze verbunden mit regelmäßigem Austausch und engen Abstimmungsprozessen zwischen den verschiedenen Kompetenzen, die sich auf dem Boden des biopsychosozialen Modells als wirksam und kosteneffektiv in der Behandlung chronischer Schmerzen gezeigt haben [18]. Ähnlich wie im (teil-)stationären Bereich sind auch für die ambulante multimodale Therapie die strukturierte Vernetzung und die definierte Kooperation mit verschiedenen Leistungsanbietern sowie ein Gruppensetting anzustreben, wenn nötig ergänzt durch individuelle Einzeltherapie [19].

### Limitationen

Die vorliegende Befragung hat zu berücksichtigende methodische Limitationen wie alle derartigen Befragungen.

Ursprünglich war geplant, alle Ärztinnen und Ärzte, die in Deutschland an der QSV nach § 135 Abs. 2 SGB V teilnehmen, zu befragen. Eine Liste der betreffenden Personen konnte jedoch von der Kassenärztlichen Bundesvereinigung oder den jeweiligen Kassenärztlichen Vereinigungen mit Hinweis auf den Datenschutz nicht zur Verfügung gestellt werden. Z. B. war es nicht möglich, die bayerischen QSV-Schmerztherapeuten über die Arztsuche der KV Bayern zu ermitteln, deren Befragung möglicherweise zu einem anderen Gesamtergebnis geführt hätte. Andererseits unterscheidet sich die Anzahl der QSV-Schmerztherapeuten in Bayern (*n* = 176) nicht wesentlich von derjenigen in Baden-Württemberg (*n* = 171; [4]). Der Zeitraum zwischen dem Auftreten erster Symptome und dem Beginn schmerztherapeutischer Maßnahmen liegt in Bayern bei 3,52 und in Baden-Württemberg bei 3,47 Jahren, im Durchschnitt aller KV-Bereiche bei 3,97 und im Durchschnitt unserer repräsentativen Stichprobe bei 3,86 Jahren [4].

Über die Arztauskunft der KV-Bezirke konnten demgemäß nur ca. 50 % der QSV-Adressen ausfindig gemacht werden. Daher wurde, um einen Selektionsbias auf dieser Ebene zu vermeiden, statt einer Vollerhebung in Deutschland eine an geografischen Gesichtspunkten orientierte Befragung vorgenommen. Inwieweit der Rücklauf in unserer Befragung von 35,9 % Aussagen zulässt, die verallgemeinerbar sind, kann nicht abschließend erschlossen werden. Allerdings gibt es bislang keine Befragung, die sich gezielt an QSV-Therapeuten gerichtet hat. Im Gegensatz zu den Umfragen des BVSD 2019 und 2012 [4, 17], in denen verschiedene an der schmerztherapeutischen Versorgung beteiligte Berufsgruppen befragt wurden, wurde in der vorliegenden Befragung durch eine Zusatzfrage die Teilnahme an der QSV verifiziert (Abb. 1).

Trotz sorgfältiger Evaluation konnte nicht jedem identifizierten QSV-Therapeuten eine gültige E‑Mail-Adresse zugeordnet werden (Tab. 8). Andererseits bietet die internetbasierte Befragung den Vorteil einer zeit- und ortsunabhängigen Befragung [20]. Dies ermöglicht eine höhere Flexibilität für die Befragten im Gegensatz zu einem telefonisch durchgeführten Interview, bei dem der Zeitpunkt der Befragung vom Interviewer bestimmt wird. Da es bei der Befragung primär um die subjektive Einschätzung der Befragten ging, war Anonymität ein wichtiges Kriterium. Internetbasierte Befragungen führen im Allgemeinen zu einem offeneren Antwortverhalten unabhängig von sozialer Erwünschtheit [21].

**Table 8:** 

Bundesland	Befragt	QSV-Teilnehmer insgesamt (Stand 2016; [4])	Befragungsquote (in %)
Berlin	28	71	39,4
Niedersachsen	45	51	86,7
Sachsen	53	87	61,0
Baden Württemberg	119	171	69,5

Insgesamt konnte mit 35,9 % eine gute (bereinigte) Rücklaufquote erzielt werden. Für Sachsen lag die Rücklaufquote allerdings nur bei 7,6 %. Dies kann nicht als repräsentativ gewertet werden. Warum die Rücklaufquote für Sachsen so niedrig war, bleibt unklar.

Da es sich bei der Befragung um subjektive Meinungen und Einschätzungen der Befragten handelt, können keine objektiven Aussagen über die derzeitige Versorgungssituation in der Schmerzmedizin getroffen werden. Hierzu bedarf es einer gesonderten Untersuchung.

## Schlussfolgerung

Bei regional sehr unterschiedlicher schmerztherapeutischer Versorgungsdichte tragen zusammenfassend die zu geringe inhaltliche und formale Eigenständigkeit der Schmerzmedizin in Deutschland sowie unzureichend funktionierende Netzwerke aus Schmerz‑, Physio- und Psychotherapeuten zur beruflichen Unzufriedenheit von Schmerztherapeuten bei. Nur ca. 40 % der niedergelassenen Schmerzmediziner bilden aus, im Gegensatz zu ca. 90 % im universitären Bereich. Eine Nachfolge nach Ausscheiden aus dem Berufsleben wird im niedergelassenen Bereich nur von etwa der Hälfte der Befragten für wahrscheinlich gehalten. Aus Sicht der hochgradig spezialisierten Schmerztherapeuten, die nahezu ausschließlich schmerztherapeutisch tätig sind, kann die Entwicklung der Schmerzmedizin in Richtung einer größeren Eigenständigkeit, wie die Einführung einer eigenen Facharztgruppe, ein möglicher Lösungsweg sein, auch zur Überwindung des akuten Nachwuchsproblems.

## Footnotes

1 Dusch, M. (2020) Persönliche Mitteilung.
